# Modelling of Pore Collapse during Polymer Sintering: Viscoelastic Model with Enclosed Gas

**DOI:** 10.3390/ma14092182

**Published:** 2021-04-24

**Authors:** Florian Wohlgemuth, Dirk Lellinger, Ingo Alig

**Affiliations:** 1 Division Plastics, Fraunhofer Institute for Structural Durability and System Reliability (LBF), Schlossgartenstr. 6, 64289 Darmstadt, Germany; florian.wohlgemuth@fmt.fau.de (F.W.); dirk.lellinger@lbf.fraunhofer.de (D.L.); 2 Institute of Manufacturing Metrology (FMT), Friedrich Alexander University Erlangen-Nürnberg, Nägelsbachstraße 25, 91052 Erlangen, Germany

**Keywords:** polymer sintering, pore collapse, coalescence dynamics, viscoelastic flow, surface tension, gas transport

## Abstract

Frenkel’s model for the late stage of coalescence of viscous particles has been extended to describe pore collapse in a viscoelastic melt during polymer sintering. The shrinkage of a pore in a polymer melt driven by surface tension is extended by taking into account the effects of trapped gas and gas transport out of the pore. Viscoelasticity has been shown to have a considerable impact on the time scale of the coalescence process. In addition, gas diffusion modifies the coalescence dynamics. Based on a parameter study, different regimes for the pore collapse have been identified. At the beginning of pore collapse, surface tension is considerably stronger than gas pressure within the pore. In this time interval (surface-tension-driven regime), the pore shrinks even in the absence of gas diffusion through the matrix. In the absence of gas transport, the shrinkage dynamic slows down and stops when the surface tension balances the gas pressure in the pore. If gas transport out of the pore is possible, surface tension and gas pressure are balanced while the gas pressure slowly decreases (diffusion-controlled regime). The final phase of pore collapse, which occurs when the gas pressure within the pore decreases sufficiently, is controlled again by surface tension. The limitations of the model are discussed. To analyze the interplay between different mechanisms and process steps during selective laser sintering, the respective time scales are compared using experimental data.

## 1. Introduction

Due to the viscoelastic nature of polymeric materials, polymer sintering differs significantly from the sintering of metals or simple viscous materials. Polymer sintering can be described as a process where polymer particles are fused together by heat or pressure. It plays an important role for several polymer-manufacturing processes, such as rotational molding, additive manufacturing (AM) or the powder coating process. In the last decade, sintering of polymer powder attained an increasing interest due to the advances of additive manufacturing processes (see, e.g., [1] and references therein), such as selective laser sintering (SLS). Sintering is also a key mechanism in latex film formation from colloidal aqueous dispersions [2]. For the understanding of these applications, a model including viscoelasticity on the microscopic level, which has a considerable impact on the time scale of the coalescence process, is essential. A serious problem of polymer sintering is the formation of trapped gas bubbles (also named pores or voids), because it can reduce the mechanical performance of the parts. Moreover, bubbles and pit holes at the surface pose problems to the part or coating appearance and aesthetics.

As early as 1945, Frenkel [3] gave two analytical solutions for the limiting cases of particle coalescence by a Newtonian viscous flow driven by surface energy minimization: (i) early stage of coalescence describing the coalescence of two spherical particles and (ii) late stage, which described the collapse of a spherical pore inside a (infinite) surrounding melt. Frenkel´s description was based on a balance between the work done by surface tension and the work by viscous dissipation. For the early stage model by Frenkel, various extensions exist; most notably the one by Bellehumeur et al. [4] modeling a viscoelastic surrounding melt by using the convected Maxwell model [5]. This model was expected to predict the general features of the coalescence of spherical polymeric particles [6]. Scribben et al. [7] continued the work of Bellehumeur et al. [4] by describing the transient viscoelastic coalescence of two particles using the upper convected Maxwell constitutive model. They compared their simulations using their model with experiments on isotactic polypropylenes and showed that the model improves the accuracy at short time scales, but does not decrease the error at long time scales. The deviations between using single- and multimode relaxation times were found to be small [7].

For the late stage of pore collapse, Mackenzie and Shuttleworth [8] developed a model extension accounting for the effect of trapped gas within the pore. This model considered a Bingham solid or a Newtonian viscous melt as the surrounding medium, but did not include viscoelasticity. A model for the sintering of polymer particles during rotational molding from Kontopoulou et al. [9] considered the diffusion of the trapped gas through the surrounding melt. In addition, there are various approaches for the description of other technical problems based on the interplay of viscous flow and surface tension such as foaming. A review of these papers can be found, e.g., in [10]. However, the parallels will not be discussed here.

The objective of the current research is to present a study on pore collapse in viscoelastic polymer melts taking into account the gas in the pore and its release by diffusive transport. Parts of the results have been presented before in two conference papers [11,12]. This paper extends the results by a more detailed discussion of the model, especially considering the gas diffusion aspects and the inclusion of ambient pressure. The discussion of transition times is considerably extended and analyzed over regions of the phase space. The discussion of the approximations resulting in limitations of the model as well as the discussion of the parameter values and time regimes of relevant processes is new. Based on Frenkel’s late stage model, an attempt has been made to develop a rigorous model, which includes the parameters relevant to the removal of bubbles from polymer melts, such as surface tension, viscoelastic relaxations and gas transport. For this reason, the viscoelastic constitutive equation used by Bellehumeur et al. [4] for description of the particle coalescence in the early stage and Mackenzie and Shuttleworth’s modifications for trapped gas [8] are combined. Motivated by the model of Kontopoulou et al. [9] and Gogos [13], gas transport out of the pore is included into the viscoelastic late stage model. By a parameter study, three different regimes of the gas pore collapse are identified. 

The paper is organized as follows: after a short description of the existing models for particle coalescence, the viscoelastic extension of the late stage model using a modified Maxwell fluid is presented. Dimensionless variables and parameters allow scaling of the results of the numerical simulation. The results are compared to Frenkel´s late stage model [3] and with the limiting case of a single, gas-filled pore in a Newtonian liquid from Mackenzie and Shuttleworth’s theory [8].

In a following section, effects of gas transport are included in the simulations by using a simplified model for the gas transfer out of the pore. A full solution in the form of a memory integral is given in Appendix A, while the main section focuses on the solution and analysis of a transfer coefficient approximation.

Subsequent to the numerical results, the approximations contained in the model and its resulting limitations are discussed. Moreover, the time and temperature regimes of different relevant physico-chemical processes during polymer laser sintering are gauged based on experimental data. The article is concluded with a summary and outlook.

## 2. Models

In Section 2.1, the models of Frenkel as well as Mackenzie and Shuttleworth are reviewed and a short outline of their ideas is given. A viscoelastic extension of the Mackenzie and Shuttleworth model for the pore collapse is presented in Section 2.2. In Section 2.3, dimensionless variables are introduced to facilitate numerical analysis, and the solutions to the models of Frenkel and Mackenzie and Shuttleworth are provided in these variables. This allows for a comparison of different models, including the viscoelastic model presented in this paper. In Section 2.4, an extension of the viscoelastic model to include diffusive gas transport out of the inclusions is presented.

### 2.1. Models of Frenkel and Mackenzie and Shuttleworth

**Figure 1:**
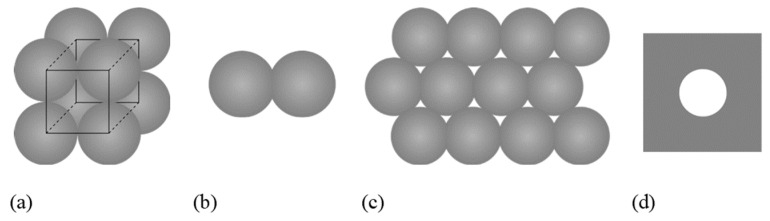
Schematic representation of coalescence of an array of spheres (**a**). In the first stage (**b**), the spherical particles coalesce and form trapped voids in between them (**c**). These voids become spherical and finally collapse (**d**).

Frenkel’s idea [3] was to idealize the sintering of a mixture of small metallic particles as the sintering of an array of spheres (Figure 1a). In the first stage, the relevant process is the sintering of adjoining spherical particles (Figure 1b). Frenkel described this as the sintering of two spheres initially in contact at exactly one point. The dynamics is driven by the minimization of surface energy—since two spheres have a larger surface than one sphere of equal volume—and slowed down by the viscosity of the melt. In an intermediate stage, which is not described by Frenkel, the spherical particles have coalesced so much that they form trapped voids in between them (Figure 1c). These voids become spherical in order to minimize surface energy. In the final stage of Frenkel’s model, the spherical pores trapped inside the metallic melt collapse (Figure 1d). This is described by the modeling of one spherical pore in an infinite viscous melt.

The driving mechanism of this process is again the minimization of surface energy. At the same time, the incompressible, infinite, homogeneous, Newtonian viscous melt surrounding the pore needs to flow in a radial direction and this flow dissipates the energy gained by surface reduction. The pore collapses linearly in time.

Frenkel already remarks that trapped gas inside the pore prevents the pore from collapsing completely. The final equilibrium radius of the pore can be deduced from a force balance between surface tension and gaseous pressure. However, Frenkel’s model does not take into account the effect of trapped gas in his late stage model. This is done by Mackenzie and Shuttleworth [8] by calculating the necessary work done onto the gas to compress it under the assumption of ideal gas behavior. In their model, the energy gained by surface reduction is now split between the work necessary to compress the gas and the energy dissipated by viscous flow. This leads to a nonlinear time dependence of the process. Mackenzie and Shuttleworth [8] further used an effective contribution volume approach to account for the effect of multiple trapped pores. This approach is not followed here. The model proposed below considers the case of a single trapped pore.

### 2.2. Viscoelastic Model

The model considers a gaseous spherical pore surrounded by an infinite, homogeneous, isotropic viscoelastic melt under isothermal conditions. Assuming incompressibility (and thus volume conservation), the radial velocity profile in the melt is given by:

(1) $$\dot{r}=\frac{r_{p}^{2}\dot{r_{p}}}{r^{2}}\Rightarrow \dot{\varepsilon }=\frac{\partial \dot{r}}{\partial r}=-2\;\frac{r_{p}^{2}\dot{r_{p}}}{r^{3}}.$$

Here $r_{p}$ is the radius of the pore, ${\dot{r}}_{p}$ the radial velocity of the pore surface and r the canonical radial coordinate of spherical coordinates within the surrounding melt.

For volume conservation to hold, the strain rate tensor D needs to have a vanishing trace [14]. Therefore, two additional flows of half magnitude and opposite sign in the angular directions are assumed. This is called the Eshelby correction [15] and is used throughout all later works based on Frenkel’s model, e.g., [4,8]. This yields for D:

(2) $$D=\left(\begin{array}{ccc} -\dot{\frac{\varepsilon }{2}} & 0 & 0\\ 0 & \dot{\varepsilon } & 0\\ 0 & 0 & -\dot{\frac{\varepsilon }{2}} \end{array}\right).$$

As a constitutive equation to model the polymeric behavior of the melt, the convected Maxwell model (CM) used by Bellehumeur et al. [4] is applied also in this work.

(3) $$\lambda \overset{□}{\tau }+\tau =2\eta D$$

Here, τ is the stress tensor, λ the relaxation time and η the viscosity. $\overset{□}{\tau }$ is a generalized objective time derivative as defined in [5].

(4) $$\overset{}{\tau }=\frac{D\tau }{Dt}-W\tau +\tau -\alpha \left(D\tau +\tau D\right);\;\alpha \in \left\{0;1;-1\right\}$$

The different values of α in Equation (4) correspond to the upper, lower and co-rotational derivative, which are equal to −1, 1 and 0, respectively. The upper convected Maxwell model (UCM) is usually applied in rheology, because it can be derived from the Rouse theory and it gives a more realistic prediction for the normal stresses in shear flow, than the lower convected Maxwell model (LCM). Since extensional flow is dominating in sintering, both UCM and LCM will be included in the following derivations. Due to the scale separation of characteristic material times and those of the process, quasi-steady flow is assumed as a first approximation following Bellehumeur et al. [4] and the time derivative Dτ/Dt is thus set to zero. The work dissipated by viscoelastic flow per time can then be calculated to be:

(5) $$\dot{W_{v}}=\int^{\;}dV\;\sum_{i,j=1}^{3}\tau_{ij}D_{ji}=\frac{8\pi }{3}\frac{\eta \dot{r_{p}}r_{p}^{2}}{\alpha \lambda }\ln\left(\frac{1+\frac{4\alpha \lambda \dot{r_{p}}}{r_{p}}}{1-\frac{2\alpha \lambda \dot{r_{p}}}{r_{p}}}\;\right).$$

The work rate done to compress the gas inside the pore can be calculated according to:

(6) $${\dot{W}}_{p}=-pS\dot{r_{p}}.$$

Here, p is the gaseous pressure and S the surface of the pore. Neither the models by Frenkel nor the one by Mackenzie and Shuttleworth consider the infinitely extended melt to be at nonzero pressure. In this paper, this assumption is retained even if it is not fulfilled for real technical processes. The inclusion of ambient pressure that is assumed to be equal to the initial gas pore pressure can however be shown to have a small effect for interesting parameter ranges, see below. The change in surface energy can be obtained according to:

(7) $${\dot{W}}_{s}=\;\sigma \dot{S\;}=8\;\pi r_{p}\dot{r_{p}}\sigma .$$

σ is the surface tension at the gas–melt interface.

For energy conservation to hold, the work rates need to add up to zero $\left(\dot{W_{v}}+{\dot{W}}_{p}+{\dot{W}}_{s}=0\right)$. For more compact notation, the abbreviation $G\left(r_{p}\right)$ is defined as:

(8) $$G\left(r_{p}\right)=\exp\left(-3\;\frac{\alpha \lambda }{\eta r_{p}}\left(\sigma -\frac{p_{0}r_{p}{\left(0\right)}^{3}}{2r_{p}^{2}}\right)\right)\;.$$

Assuming ideal gas behavior, energy conservation yields a differential equation for $r_{p}$:

(9) $$\dot{r_{p}}=\frac{r_{p}}{2\alpha \lambda }\frac{G\left(r_{p}\right)-1}{2+G\left(r_{p}\right)}\;\;$$

### 2.3. Dimensionless Variables

A dimensionless variable can be obtained by dividing the original variable by a characteristic value, such as the initial pore radius or a characteristic time for the pore collapse. Analyzing a model in terms of dimensionless variables has the benefit of identifying parameter sets that lead to the same generalized behavior. Here, the following dimensionless variables are introduced to facilitate numerical analysis of the model:

(10) $$\begin{array}{l} {\tilde{r}}_{p}=\frac{r_{p}}{r_{0}}\\ \tilde{t}=\frac{1}{2}\frac{\sigma }{r_{0}\eta }t=\frac{t}{\tau_{Frenkel}}\\ {\tilde{r}}_{p,eq}=\sqrt{\frac{p_{0}r_{0}}{2\sigma }}\\ \tilde{\lambda }=-\frac{\alpha \lambda \sigma }{2r_{0\;}\eta }=-\frac{\alpha \lambda }{\tau_{Frenkel}}. \end{array}$$

The dimensionless pore radius ${\tilde{r}}_{p}\;$is the ratio of the actual pore radius $r_{p}\left(t\right)$ divided by its initial value $r_{0}=r_{p}\left(t=0\right)$ before sintering.

The dimensionless time $\tilde{t}$ is defined via the Frenkel model. Using Frenkel’s solution for the pore collapse [3], which is given by

(11) $$r_{p}\left(t\right)=r_{0}-\frac{1}{2}\frac{\sigma }{\eta }t,$$

one can define a characteristic time $\tau_{Frenkel}=2\;r_{0}\;\eta /\sigma$, at which a pore embedded in a simple Newtonian fluid disappears $\left(r_{p}=0\right)$ in the Frenkel model (neglecting the embedded gas). Dividing the actual time t by $\tau_{Frenkel}$, a dimensionless time $\tilde{t}$ can be introduced.

Gas inside a shrinking pore needs to be compressed for the pore to shrink. This will result in an increase in gas pressure inside the pore. Assuming ideal gas behaviour, the pressure increase can be predicted and the radius at which gas pressure balances surface tension can be derived from a simple balance of forces. This (dimensionless) equilibrium radius is called ${\tilde{r}}_{p,eq}$. This equilibrium radius is a steady state value for any isotropical incompressible viscoelastic melt [16].

Finally, a dimensionless relaxation time $\tilde{\lambda }$ is defined as the characteristic time of the viscoelastic relaxation (αλ) divided by the time of a simple viscous pore collapse ($\tau_{Frenkel}$). Consequently, $\tilde{\lambda }\;$is a measure of the relation between the time needed for the rearrangements or disentanglements of the polymeric chains and an idealized pore collapse in Newtonian liquid (neglecting the pressure of trapped gas). As an approximation for the relaxation time λ, often the longest relaxation time in the distribution is taken. In analogy to Balemans et al. [17], who reported on a computational approach for the sintering of two viscoelastic particles, $\tilde{\lambda }$ can be considered a Deborah number (*De*). A Deborah number is the ratio of the time scale of the fluid response to that of the considered process. The former is here the viscoelastic relaxation time, whereas the latter is assumed to be the viscous pore collapse (for details see Section 3.3, below).

Using these quantities, the prediction of the Eshelby-corrected Frenkel model shows a universal behavior:

(12) $${\tilde{r}}_{p}\left(\tilde{t}\right)=1-\tilde{t}.$$

The model by Mackenzie and Shuttleworth [8] for one single pore in dimensionless variables yields the following differential equation:

(13) $$\frac{d{\tilde{r}}_{p}}{d\tilde{t}}=-1+\frac{{\tilde{r}}_{p,eq}{}^{2}}{{\tilde{r}}_{p}{}^{2}}.$$

The differential equation for the viscoelastic (VE) model presented in this work reads in the dimensionless variables:

(14) $$\frac{d{\tilde{r}}_{p}}{d\tilde{t}}=\frac{{\tilde{r}}_{p}}{2\;\tilde{\lambda }}\;\frac{1-\exp\left(6\;\frac{\tilde{\lambda }}{{\tilde{r}}_{p}}\left(1-\frac{{\tilde{r}}_{p,eq}{}^{2}}{{\tilde{r}}_{p}{}^{2}}\right)\right)\;}{2+\exp\left(6\;\frac{\tilde{\lambda }}{{\tilde{r}}_{p}}\left(1-\frac{{\tilde{r}}_{p,eq}{}^{2}}{{\tilde{r}}_{p}{}^{2}}\right)\right)\;}\;$$

### 2.4. Gas Transport into the Polymer Matrix

If gas is trapped in a pore surrounded by a polymer melt, the gas will also be able to diffuse into the surrounding polymer matrix. For this effect to be included in the model, the gas pore pressure needs to be considered as a new time dependent variable. The rate of compression work (compare Equation (6)) now needs to be written as:

(15) $${\dot{W}}_{p}=-4\pi r_{p}^{2}p\left(t\right)\dot{r_{p}}.$$

We will below express this work rate in terms of dimensionless variables. For this purpose, the dimensionless pressure is introduced as $\tilde{p}=p\left(t\right)/p_{0}$, where $p_{0}$ is the initial pressure inside the pore at t=0.

Assuming that diffusion and diffusive transfer across the liquid–gas boundary can be described by Fick’s law with diffusion coefficient D, the mass balance for the gas yields a differential equation for the pore pressure (Equation (16), compare [9]). T stands for the temperature and R for the universal gas constant.

(16) $$\begin{array}{l} \frac{4}{3}\pi \frac{d}{dt}\left(\frac{p\left(t\right)r_{p}^{3}}{RT}\right)=\left(4\pi r_{p}^{2}\right)D{\left(\frac{\partial c\left(r,t\right)}{\partial r}\right)}_{r=r_{p}}\;\\ \Rightarrow \frac{dp\left(t\right)}{dt}=\frac{1}{r_{p}}\left(3RTD{\left(\frac{\partial c\left(r,t\right)}{\partial r}\right)}_{r=r_{p}}-3p\left(t\right)\;{\dot{r}}_{p}\right) \end{array}$$

The concentration of the gaseous species inside the melt can be described using the equations of Fickian diffusion in moving media (compare [18]):

(17) $$\frac{\partial \tilde{c}}{\partial \tilde{t}}+\frac{d{\tilde{r}}_{p}}{d\tilde{t}}\frac{{\tilde{r}}_{p}{}^{2}}{{\tilde{r}}^{2}}\;\frac{\partial \tilde{c}}{\partial \tilde{r}}={\left(\frac{d\tilde{t}}{dt}\right)}^{-1}\frac{D}{r_{0}{}^{2}}\;\frac{1}{{\tilde{r}}^{2}}\frac{\partial }{\partial \tilde{r}}\left({\tilde{r}}^{2}\frac{\partial \tilde{c}}{\partial \tilde{r}}\right)$$

$\tilde{r}$ and $\tilde{c}$ are the dimensionless radial coordinate and concentration, defined via $\tilde{r}=r/r_{0}$ and $\tilde{c}=c/c_{i}$. $c_{i}$ is the initial gas concentration of the melt. The dimensionless radial coordinate is in units of the initial pore radius.

The concentration at the gas–liquid interface can be obtained using Henry’s law (with dimensionless Henry constant ${\tilde{K}}_{h}$ and dimensionless pressure $\tilde{p}=p/p_{0}$) [9]. If the normal Henry constant relating concentration and pressure is given by $K_{h}$, the reduced constant is given by ${\tilde{K}}_{h}=K_{h}\cdot p_{0}/c_{i}$.

(18) $$\begin{array}{l} \tilde{c}\left({\tilde{r}}_{p},\tilde{t}\right)={\tilde{K}}_{h}\tilde{p}\\ \tilde{c}\left(\tilde{r},0\right)=1\;\mathrm{for}\;\tilde{r}>{\tilde{r}}_{p} \end{array}$$

The analytical solution to this problem can be found in Appendix A. In the following, for the sake of simplicity and a lower computation time for the study of the parameter-dependent behavior of the model, an effective gas transfer coefficient approximation is used instead of a full solution of Fick’s law. The assumptions of this approximation are that the species transfer out of the gas pore is proportional to the product of interface surface, a gas transfer coefficient (dimensionless coefficient: ${\tilde{k}}_{trans}$) and the concentration difference between both phases. The concentration in the melt is further assumed to be at a negligible concentration (approximately zero), therefore the concentration difference is given by Henry’s law. A diffusive concentration profile is not simulated—it is assumed that all the gas diffusing across the boundary is transported into the melt sufficiently fast such that there is no concentration buildup around the pore. This means that the diffusion-related transport term in Equation (16) is approximated according to Equation (19).

(19) $$\frac{3RTD}{r_{p}}{\left(\frac{\partial c\left(r,t\right)}{\partial r}\right)}_{r=r_{p}}\approx \frac{r_{0}}{{\tilde{r}}_{p}}\cdot {\tilde{k}}_{trans}\cdot {\tilde{K}}_{h}\tilde{p}=\tilde{g}\cdot \frac{\tilde{p}}{{\tilde{r}}_{p}}$$

In Equation (19), a new dimensionless parameter $\tilde{g}$ is defined as$\;\tilde{g}=\frac{{\tilde{k}}_{trans}{\tilde{K}}_{h}}{r_{0}}$. The magnitude of $\tilde{g}$ is a measure for how quickly gas will diffuse out of the pore.

Using this approximation, the differential equation for the gas pore pressure (Equation (16)) is considerably simplified and reads:

(20) $$\frac{d\tilde{p}}{d\tilde{t}}=-\frac{\tilde{p}}{{\tilde{r}}_{p}}\left(3\frac{d{\tilde{r}}_{p}}{d\tilde{t}}+\tilde{g}\right).$$

The differential equation for the dimensionless pore radius results again from imposing energy conservation $\left(\dot{W_{v}}+{\dot{W}}_{p}+{\dot{W}}_{s}=0\right)$ using the modified expression for ${\dot{W}}_{p}$ (Equation (15)).

(21) $$\frac{d{\tilde{r}}_{p}}{d\tilde{t}}=\frac{{\tilde{r}}_{p}}{2\tilde{\lambda }}\frac{1-G_{2}\left({\tilde{r}}_{p},\;\tilde{p}\left(\tilde{t}\right)\right)}{2+G_{2}\left({\tilde{r}}_{p},\;\tilde{p}\left(\tilde{t}\right)\right)}\;\;\;G_{2}\left({\tilde{r}}_{p},\;\tilde{p}\left(\tilde{t}\right)\right)=\exp\left(\frac{6\tilde{\lambda }}{{\tilde{r}}_{p}}\left(1-{\tilde{r}}_{p}{\tilde{r}}_{p,eq}{}^{2}\tilde{p}\left(\tilde{t}\right)\right)\right)\;.$$

## 3. Modelling of Pore Collapse—Numerical Results

In Section 3.1, the numerical results of our viscoelastic model are presented in comparison to the models of Frenkel and Mackenzie and Shuttleworth. The numerical results for the inclusion of gas diffusion into the viscoelastic model are presented in Section 3.2. In Section 3.3, characteristic time regimes for the pore collapse are defined and presented on “collapse mechanism maps”. The dependence of the duration of these regimes is discussed in terms of the dependence of the control parameters, such as viscoelastic relaxation time, gas transfer coefficient and equilibrium pore radius. Section 3.4 extends this discussion by simulations and the representation of the duration of the diffusion-controlled regime depending on parameter settings. This regime is intermediate between two surface tension-controlled regimes.

### 3.1. Viscoelastic Model

**Figure 2:**
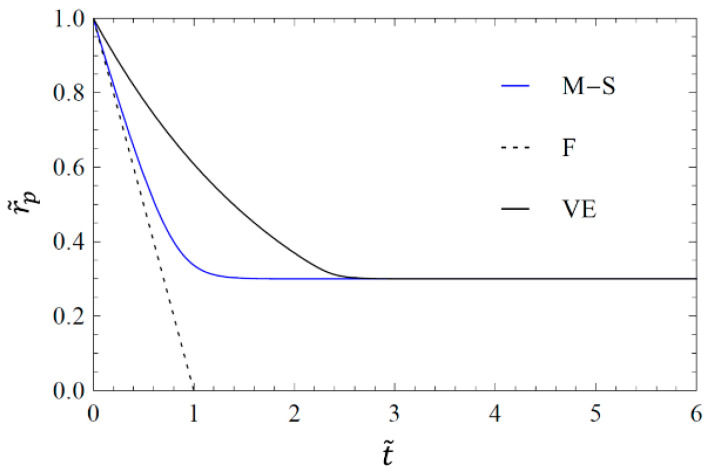
Solution curves for the Frenkel model (F), the (modified) Mackenzie and Shuttleworth model (M–S) and the viscoelastic model described in this paper (VE) for parameter values of ${\tilde{r}}_{p,eq}=0.3$ and $\tilde{\lambda }\;=\;1$.

The dimensionless radius ${\tilde{r}}_{p}$ as a function of dimensionless time for the Frenkel model (F), the (modified) Mackenzie and Shuttleworth model (M–S) and the viscoelastic model (VE) are compared in Figure 2. Equations (13) and (14) were solved numerically using Wolfram’s Mathematica [19].

For most of the simulations presented in this study, α was set to −1, which refers in rheology to the upper convected Maxwell (UCM) model. The linear decrease in Frenkel’s model shows a pronounced difference from the dynamics shown by the other two models. Both the model developed in this work and the Mackenzie and Shuttleworth model show an equilibrium radius ${\tilde{r}}_{p,eq}$. The model of Mackenzie and Shuttleworth does however collapse to this equilibrium radius more quickly due to the Newtonian viscous character of the melt.

**Figure 3:**
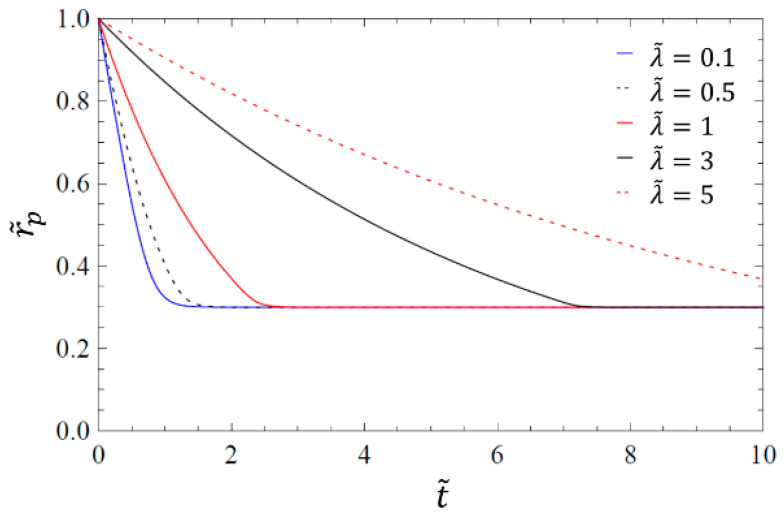
Solution curves for the viscoelastic model described in this paper for a constant equilibrium radius ${\tilde{r}}_{p,eq}=0.3$ and various relaxation times as indicated by the legend (adapted from [12]).

Figure 3 shows solutions of the model described in this work for different dimensionless relaxation times $\tilde{\lambda }$. It can be seen that a higher relaxation time leads to longer process dynamics.

If the ambient pressure of the surrounding melt is included, the energy work rate against the gaseous pressure (Equation (6)) is modified:

(22) $${\dot{W}}_{p}=-\left(p-p_{\mathrm{melt}}\right)S\dot{r_{p}}.$$

Here, $p_{\mathrm{melt}}$ is the ambient pressure of the surrounding melt.

**Figure 4:**
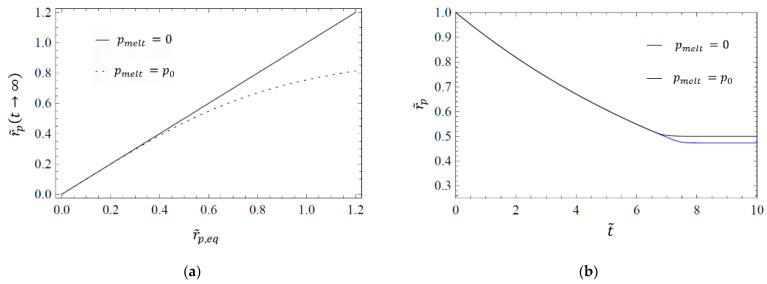
Comparison of the equilibrium radius ${\tilde{r}}_{p,eq}$ (at t→∞) as a function of the equilibrium value of the pore diameter (**a**) and the pore collapse dynamics (**b**), with and without consideration of the hydrodynamic ambient pressure acting on the surrounding melt $p_{\mathrm{melt}}$. The parameters for both plots are ${\tilde{r}}_{p,eq}=0.5$ and $\tilde{\lambda }=5$.

As a result of the inclusion of ambient pressure, the model exhibits a different equilibrium radius. A comparison between equilibrium radius with and without ambient pressure can be seen in Figure 4a. For low values of ${\tilde{r}}_{p,eq}$, the deviation is not drastic. When the pore becomes compressed, the gas inside the pore also becomes compressed, and thus the inner gas pressure raises. For low equilibrium radii, and thus considerable compression of the pore, this increase in gas pressure is substantial, and the ambient pressure becomes negligible in comparison. Only if there is little compression, and thus little increase in gas pressure inside the pore (thus for high values of ${\tilde{r}}_{p,eq}$), the deviation is non-negligible. Figure 4b shows that the time evolution is not influenced considerably by the ambient pressure—again for the case of a relatively low ${\tilde{r}}_{p,eq}$.

### 3.2. Viscoelastic Collapse with Gas Transport (Transfer Coefficient Model)

**Figure 5:**
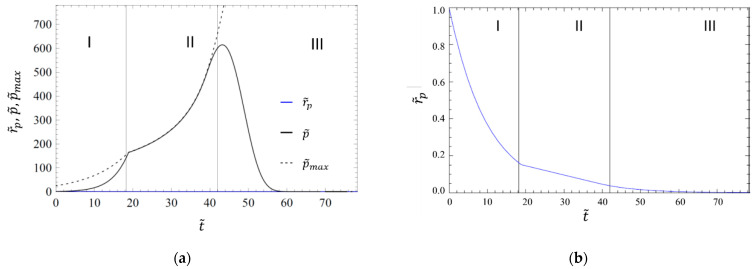
Radius ${\tilde{r}}_{p}$ (in blue), gas pressure $\tilde{p}=p/p_{0}$ in the pore (in black) and maximum gas pressure ${\tilde{p}}_{max}\;$above which the pore will expand (dashed black), as a function of dimensionless time $\tilde{t}\;$for the transfer coefficient model with $\tilde{g}=0.01$, ${\tilde{r}}_{p,eq}=0.2$ and $\tilde{\lambda }=5$ (plot (**a**)). Magnification of the blue curve for ${\tilde{r}}_{p}\;$versus $\tilde{t}$ from (**a**) (plot (**b**)) (adapted from [12]).

A plot of a solution of Equations (17) and (18) for the dimensionless gas pressure $\tilde{p}=p/p_{0}$ and the radius ${\tilde{r}}_{p}\;$is shown in Figure 5a,b. The axis for ${\tilde{r}}_{p}$ is rescaled in Figure 5b. The plot in Figure 5a includes a dotted line for the limiting (maximum) gas pressure ${\tilde{p}}_{max}$, above which the pore will expand due to the inner gas pressure. This maximum pressure is the pressure at which the local force exerted by pressure and surface tension cancel. It can be calculated to be ${\tilde{p}}_{max}=1/({\tilde{r}}_{p}{\tilde{r}}_{p,eq}{}^{2})$. Three distinct regimes are recognizable, which are separated by vertical lines in Figure 5. The regimes are denoted from left to right as regimes I, II and III. In regimes I and III, the surface tension is considerably larger than the gas pressure, and even in the absence of diffusion, the pore would shrink. These regimes are surface-tension-driven regimes. In regime II, surface tension and gas pressure are of almost equal magnitude. In the absence of gas diffusion in the surrounding polymer melt, the pore collapse dynamics would thus slow down and stop. Therefore, regime II is called the diffusion-controlled regime. The exact transition times between the regimes are to some extent arbitrary. In this work, the condition ${\tilde{p}}_{max}=1,10\;\tilde{p}$ is used to distinguish the two surface-tension-driven regimes (I and III) and the diffusion-controlled regime (II).

### 3.3. Collapse Mechanism Maps

Using the approach described above, it is possible to generate parameter maps that visualize which of the collapse regimes apply for a given set of parameters. In these maps, the different collapse mechanisms are separated by characteristic transition times between the regimes.

**Figure 6:**
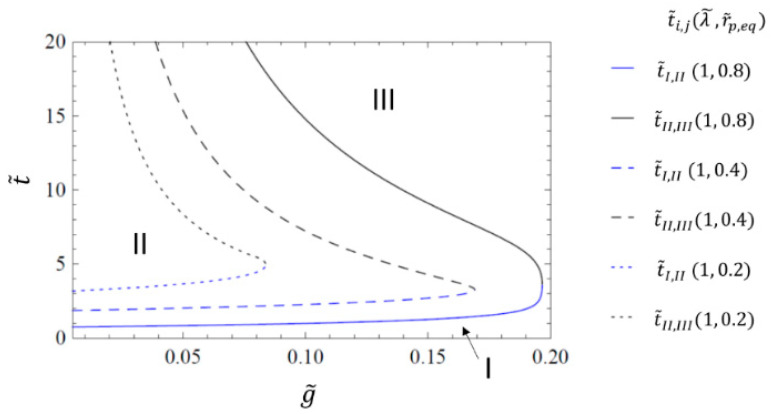
Transition times ${\tilde{t}}_{I,\;II}$ (blue lines) and ${\tilde{t}}_{II,III}\;$(black lines) as a function of gas transfer rate $\tilde{g}$ for the transfer coefficient model, with a relaxation time of $\tilde{\lambda }=1$ and different values of ${\tilde{r}}_{p,eq}$. The quantities in the legend are arranged as $\left(\tilde{\lambda },{\tilde{r}}_{p,eq}\right)$. The three pore collapse regimes are indicated by I, II and III.

The collapse mechanism map in Figure 6 shows exemplarily the transition times ${\tilde{t}}_{I,II}\;\left(\mathrm{blue}\;\mathrm{lines}\right)\;$between regimes I and II, as well as the time$\;{\tilde{t}}_{II,III}\;$(black lines) between regimes I and II as a function of the rate of gas transfer $\tilde{g}$ for different values of ${\tilde{r}}_{p,eq}$. The quantities in the legend are arranged as $\left(\tilde{\lambda },{\tilde{r}}_{p,eq}\right)$, with a fixed value of $\tilde{\lambda }=1$. Since$\;{\tilde{r}}_{p,eq}$ is proportional to $\sqrt{p_{0}}$ (compare (10)), it is proportional to the initial amount of gas inside the pore. Thus, for constant values of $\tilde{g}$, the duration of the diffusion-controlled regime II (time difference: Δ${\tilde{t}}_{II}={\tilde{t}}_{II,III}-{\tilde{t}}_{I,II}$) increases with increasing values of ${\tilde{r}}_{p,eq}$. The reason for this is that the diffusive transport of the gaseous species into the melt takes, for the same of value $\tilde{g}$, less time for a smaller initial amount of trapped gas. 

Since gas pore pressure and surface tension are balanced in regime II, the gas pressure in the pore reduces faster if the gaseous species are transported more quickly from the pore into the liquid matrix. Faster diffusion, which is related to a higher value of $\tilde{g},$ shortens the duration of the diffusion-controlled regime II, which is indicated by a smaller time difference Δ${\tilde{t}}_{II}$ for increasing values of $\tilde{g}$.

**Figure 7:**
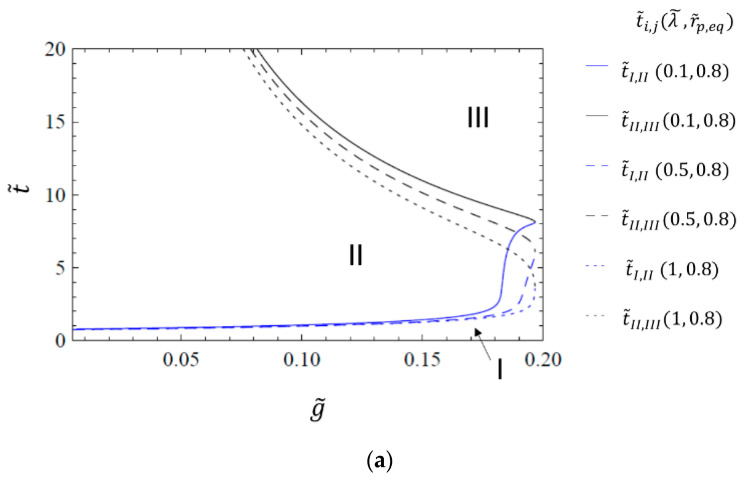

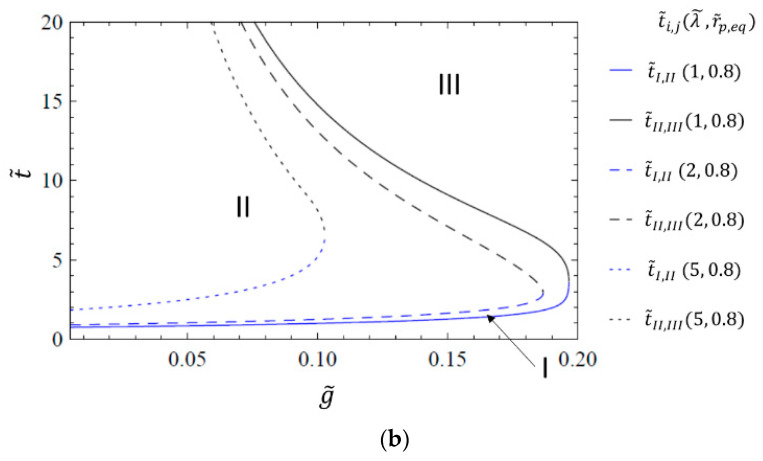
Transition times as a function of the gas transfer rate $\tilde{g}$ for the transfer coefficient model, for different values of the relaxation time $\tilde{\lambda }\leq 1$ (**a**) and $\tilde{\lambda }\geq 1$ (**b**) at a fixed value of ${\tilde{r}}_{p,eq}=0.8$. The quantities in the legend are arranged as $\left(\tilde{\lambda },{\tilde{r}}_{p,eq}\right)$.

For all three combinations of $\tilde{\lambda }$ and ${\tilde{r}}_{p,eq}$ in Figure 6, the duration of the diffusion-controlled regime II, which is expressed by Δ${\tilde{t}}_{II}$, becomes smaller for increasing values of the gas transfer rate $\tilde{g}$. Above a certain upper value of$\;\tilde{g}\;$, the diffusion-controlled regime II disappears for all combinations of $\tilde{\lambda }$ and ${\tilde{r}}_{p,eq}$ completely (see, e.g., $\tilde{g}>0.17$ for ${\tilde{r}}_{p,eq}=0.4$), becomes zero and the diffusion-controlled regime II disappears. This upper $\tilde{g}$ value depends on $\tilde{\lambda }$ and ${\tilde{r}}_{p,eq}$ and exhibits the characteristics of a critical point. This “upper critical value” of $\tilde{g}$ has, however, not been above 0.2 for any combination of ${\tilde{r}}_{p,eq}$ and $\tilde{\lambda }$ in the simulations of this study (see also Figure 7).

Figure 7 shows the dimensionless transition times$\;{\tilde{t}}_{I,II}\;$and ${\tilde{t}}_{II,III}\;$as functions of the gas transfer rate g, for different values of the relaxation time $\tilde{\lambda }$ for a fixed value of ${\tilde{r}}_{p,eq}=0.8$. The quantities in the legend are arranged as in Figure 6 as $\left(\tilde{\lambda },{\tilde{r}}_{p,eq}\right)$. For values of $\tilde{\lambda }\leq 1\;$in Figure 7a, the $\tilde{\lambda }$ values mainly influence the shape of the contour, i.e., the exact beginning and end times of the different regimes. For values of $\tilde{\lambda }\geq 1$ (see Figure 7b) the parameter range for which the pore collapse dynamics show three distinct regimes becomes smaller, since viscoelastic collapse is slower for higher relaxation times. As the diffusion sets in from the beginning, the surface-tension-driven viscoelastic pore collapse and the diffusion-controlled regime overlap more and more until no difference is visible.

### 3.4. Gas Diffusion-Controlled Region

**Figure 8:**
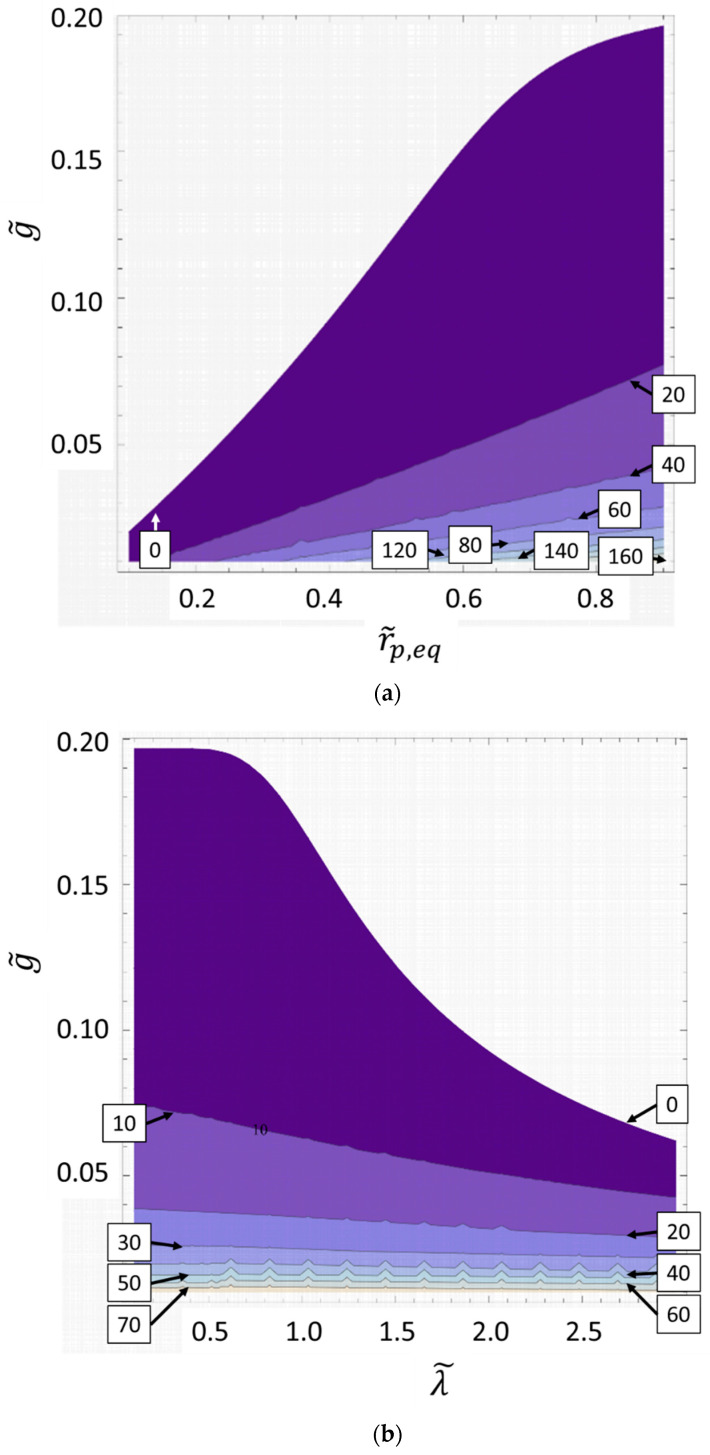

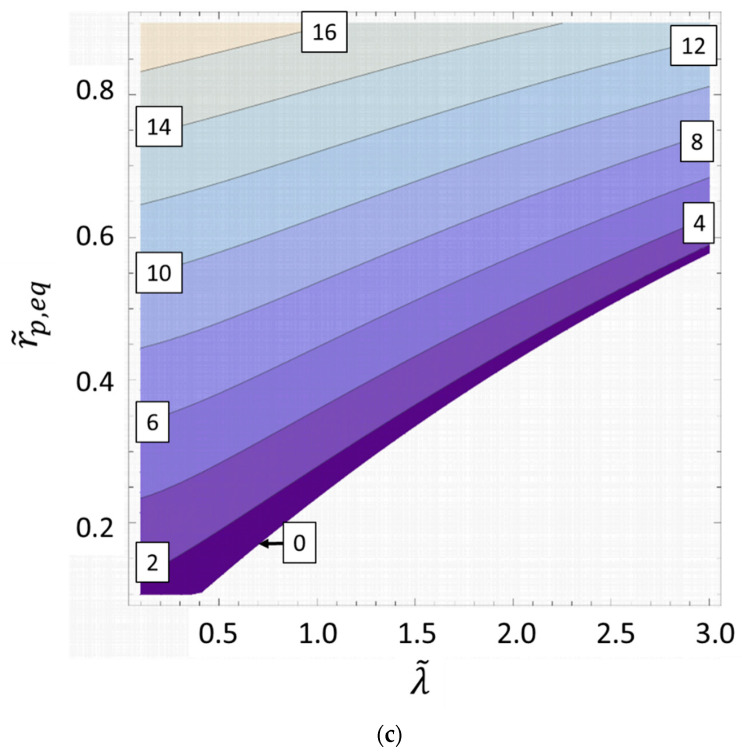
Time duration Δ${\tilde{t}}_{\mathrm{II}}$ of the diffusion-controlled regime II as a function of the relaxation time $\tilde{\lambda }$ and the gas transfer rate $\tilde{g}$ for ${\tilde{r}}_{p,eq}=0.4$ (a), the dimensionless equilibrium radius ${\tilde{r}}_{p,eq}$ and gas transfer rate $\tilde{g}$ for $\tilde{\lambda }=2$ (b), as well as ${\tilde{r}}_{p,eq}$ and $\tilde{\lambda }$ for $\tilde{g}=0.1\;\left(c\right)$. The values of Δ${\tilde{t}}_{\mathrm{II}}\;$are indicated at the contour lines. The white areas represent the absence of region II, i.e., the surface-tension-driven regions I and III merge, without a diffusion-controlled regime in between.

The time difference between both dimensionless transition times (Δ${\tilde{t}}_{II}={\tilde{t}}_{II,III}-{\tilde{t}}_{I,II}$), which is the time duration of the diffusion-controlled regime II, can be plotted as a two-dimensional contour plot by selecting two of the three parameters $\tilde{g},\;\tilde{\lambda }$ and ${\tilde{r}}_{p,eq}$, (such as $\left({\tilde{r}}_{p,eq},\tilde{g}\right)$,$\;\left(\tilde{\lambda },\tilde{g}\right)$ or $\left(\tilde{\lambda },{\tilde{r}}_{p,eq}\right)$) as variables. This permits study of the interdependence of the parameters in a compact way, as shown in Figure 8a–c.

Figure 8a shows that for low values of the gas transfer rate $\tilde{g}$, the duration of the diffusive regime is almost independent of the value of $\tilde{\lambda }$. Due to the low diffusion rate, there is always enough time for the surface-driven dynamics to reach an equilibrium point. This changes with larger values of $\tilde{g}$, i.e., with faster diffusion. It can be seen that the smaller the value of the relaxation time $\tilde{\lambda },\;$the more likely a distinct three regimes dynamics is still to happen. 

Figure 8b shows that the higher the value of the equilibrium radius ${\tilde{r}}_{p,eq}$ and the smaller the value of $\tilde{g}$, the more likely the three regimes dynamics is to occur. It also shows that a higher value of $\tilde{g}$ can be compensated for by a higher value of ${\tilde{r}}_{p,eq}$. Since ${\tilde{r}}_{p,eq}$ is proportional to the initial amount of gas inside the pore, this can be understood intuitively. In other words, the more gas is inside the pore and the slower the transport of this gas into the melt is, the more likely it is for the system to go into a steady state in which the only change occurs by further diffusion out of the pore (regime II). 

Figure 8c shows that the effect of higher relaxation time $\tilde{\lambda }$ to not produce discernible three regimes dynamics can be counterbalanced by a higher equilibrium radius ${\tilde{r}}_{p,eq}$. This is due to the fact that a higher equilibrium radius means both that the compression which can happen due to surface tension alone is smaller and thus needs less time, as well as that there is more gas inside the pore which will need to be transported out of the pore in the second diffusive regime.

## 4. Discussion with Respect to Laser Sintering

Section 4.1 summarizes the approximations of the viscoelastic model presented in this paper. Section 4.2 discusses experimental values for the different parameters and the time regimes of the different processes relevant to the technical application in the laser sintering process. For comparison of technically-relevant time scales, different Deborah numbers are proposed.

### 4.1. Limitations of the Model

The model presented above is formulated in dimensionless variables and deals with some general aspects of the pore collapse of viscoelastic particles at the final stage of sintering. Although the model is not limited to application-oriented questions of selective laser sintering (SLS) or additive manufacturing (AM) in general, it is one of the objectives of this paper to identify properties and the parameter space, which control polymer sintering. 

The assumptions and limitations of our viscoelastic model of pore collapse are:

(a) Single spherical inclusions with a defined initial sphere radius in an infinite melt.
(b) Pore collapse under isothermal conditions.
(c) Modified Maxwell fluid with one characteristic relaxation time.
(d) Pseudo-steady flow approximation in the rheological equation (here, mainly UCM).
(e) Description of the concentration at the gas–liquid interface by using Henry’s law.
(f) Transfer coefficient approximation of the gas transfer into the polymer matrix.

The relevance of these limitations of the model for description of the SLS process of polymeric materials will be discussed in the following text in detail.

Related to (a), the distribution of the powder grain size and shape (see e.g., [6,20]) results, in the late stage of sintering, in a distribution of size and diameter of the pores. Even deviations from spherical shape, such as elongated pores between the deposited fibers in the fused deposition modeling (FDM) process, occur. In addition, there are irregular gas inclusions at the boundary between the subsequently sintered layers and inhomogeneous packing of the powder, which can be significantly larger than those resulting from the interstices in between the grains. Due to the large shape and size distribution of particles and the resulting inclusions, gas can escape to the surface through open paths between them [21] before they close and form spherical bubbles. Furthermore, larger bubbles can possibly grow at the expense of smaller ones by Ostwald ripening. All this leads to great uncertainties with regards to the shape, dimension and size of the pores and their distributions, as well as the gas pressure in the pores. The approximation of an infinite melt should be reasonable as long as the pores are reasonably separated as the flow profile scales with an inverse cubic law (see Equation (1)). Following the approximation by Mackenzie and Shuttleworth [8], one can assume that each pore is surrounded by an effective shell region, which represents the significant interaction region of each pore (i.e., that all significant contributions to the energy dissipation are in this region). Let this effective shell have a radius $r_{2}$ (dimensionless radius ${\tilde{r}}_{2}=r_{2}/r_{0}$). Then, the total dissipated energy (compare Equation (5)) becomes:

(23) $$\dot{W_{v}}=\int^{\;}dV\;\sum_{i,j=1}^{3}\tau_{ij}D_{ji}=\frac{8\pi }{3}\frac{d{\tilde{r}}_{p}}{d\tilde{t}}{\tilde{r}}_{p}^{2}\frac{\eta r_{0}^{3}}{\tau_{Frenkel}^{2}}\ln\left(\frac{1+2\tilde{\lambda }\frac{d{\tilde{r}}_{p}}{d\tilde{t}}}{1-4\tilde{\lambda }\frac{d{\tilde{r}}_{p}}{d\tilde{t}}}\;\frac{1-\frac{4\tilde{\lambda }}{{\tilde{r}}_{2}^{3}}\frac{d{\tilde{r}}_{p}}{d\tilde{t}}}{1+\frac{2\tilde{\lambda }}{{\tilde{r}}_{2}^{3}}\frac{d{\tilde{r}}_{p}}{d\tilde{t}}}\right).$$

We obtain the same result as Mackenzie and Shuttleworth [8]: the additional term due to the shell scales with ${\tilde{r}}_{2}^{-3}$, as the new term in the energy dissipation is a small perturbation as long as ${\tilde{r}}_{2}^{-3}$ is small. This means that, if each pore has an effective shell of, e.g., 2.5 times its initial radius (corresponding to a pore-to-pore distance of five times its initial radius), the contribution is at 6%. We expect that this will not alter the model dynamics significantly. A full solution would be possible, but has not been attempted in this work.

In relation to (b), the most critical control parameter is the temperature. The strong temperature dependence of the zero-shear rate viscosity ($\eta_{0}=\eta$) and the viscoelastic relaxation times *λ* are of great importance for polymer sintering, because both quantities control the coalescence and pore collapse kinetics. In polymer melts, the temperature dependence of the viscosity and the relaxation times can be described by the Vogel–Fulcher (VF) or William–Landel–Ferry (WLF) equation, which diverges when the (idealized) glass transition temperature is approached. Well above the glass transition temperature, often a simple Arrhenius law holds. By infrared heating of the powder, the temperature is raised to about 10 K below the melting temperature. Although the temperature can be controlled satisfactorily in the powder bed, the heating by the laser beam with the SLS is highly non-isothermal and spatially inhomogeneous. For a laser power of 12.5 W with 0.83 m/s (equivalent to 1.4 J/cm^2^) and for 50 W with 2.5 m/s (equivalent to 2.86 J/cm^2^), Franco et al. [22] obtained, for polyamide 12, temperature rises of approximately 30 and 46 K from model calculations. These values for the energy density are comparable to the 2.5 J/cm^2^ proposed by Dupin [6,23] to be a good choice for SLS processing with a low degree of porosity. Therefore, modelling coupled temperature diffusion dynamics would be an important extension of the model.

Related to (c), as a first approximation for the characteristic material time which the viscoelastic polymer takes to adjust to the driving force of sintering, the longest time of the relaxation time spectrum of the polymer melt can be taken. This is for entangled polymer chains the reptation time, which is the time long polymer chains need to disentangle from their topological restraints, represented in the model of Doi and Edwards [24] by a virtual tube formed by the network of entangled chains that surround each polymer chain. Shorter relaxation times are the normal mode spectrum predicted by the Rouse model, and “local modes” the short-time end. It is important to remember that the characteristic relaxation times and the shape of the spectrum depend on temperature and molecular weight, as well as on the chain architecture such as branches or crosslinks. The latter can change significantly due to thermal aging by laser heating and during the long times at moderate temperatures in the powder reservoir or bed. The relevant aging mechanisms are chain scission, branching and/or crosslinking ([6,20] and references therein). 

**Figure 9:**
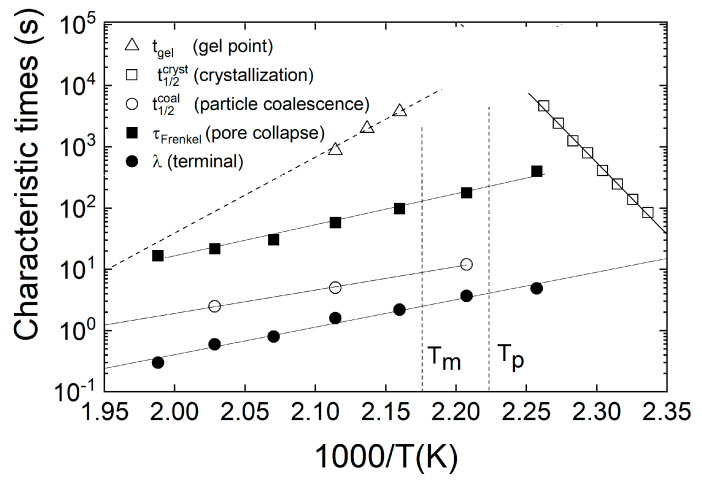
Characteristic times (see text) versus inverse temperature for the sintering of polyamide 12.

Related to (d), the limitations of the steady flow approximation become relevant if the time of the pore collapse becomes shorter than the relaxation time for the viscoelastic stress or creep. This relation can be expressed by the dimensionless Deborah number (De), which is the ratio of the time it takes for a material to adjust to applied stresses or deformations, and the characteristic time scale of the experiment or process. As a first approximation for the pore collapse one can assume$:\;De=\lambda /\tau_{Frenkel}=\lambda \sigma /(2\;r_{0}\;\eta$). As stated by Scribben et al. in [7] the steady flow approximation may not be valid for materials with large Deborah numbers, or at least in cases where the coalescence time and the longest relaxation time in the distribution are similar. For these cases, a complete transient representation is required. Possible approaches for this are discussed in [16]. Both the neck growth during the coalescence of molten polymer particles and pore collapse are very slow in a polymer melt. In processes such as SLS or rotational molding, they are at least one order of magnitude longer than the characteristic material times (see also Figure 9, below).

A full diffusion approach considering the gas absorption in the polymer melt and the substitution of Henry’s law, related to (e) and (f), remains the subject of a subsequent investigation. Some ideas for extension of the model can be found in [16].

### 4.2. Time and Temperature Regimes of Laser Sintering

As stated in the introduction, polymer sintering is inherent in industrial processes such as powder coating, dispersion coating, cold compression and rotational moulding, SLS or FDM. However, particle coalescence is only one of many mechanisms that are linked together by the process steps. In the following consideration of relevant time regimes, we concentrate on selective laser sintering. During SLS, processes such as radiative heat transfer from the laser beam to the polymer particles, inter-particle heat diffusion, the melting kinetics of grains, coalescence phenomena driven by surface tension and the release of entrapped gases, crystallization kinetics of semi-crystalline polymers and polymer aging determine the final properties of the sintered parts [6,20,21]. Each of these mechanisms has its characteristics such as length scale, time regime or temperature dependence. 

We wanted to estimate and compare the relevant time and temperature regimes for SLS, exemplarily for polyamide 12 (PA12), which is until now the most common polymer material for SLS. Therefore, we combined experimental results on characteristic times from Dupin et al. [6,23] and some of our own data [20] in an overview (Figure 9). Similar data for other polymers such as polyethylene, polypropylene and polystyrene from different sources are collected in reference [16]. However, it is very difficult to find reliable data sets for the same polymer at comparable conditions.

In Figure 9, the characteristic times are plotted as an example for PA12 in comparison to the inverse absolute temperature (1000/T (K)) in an Arrhenius diagram. To give an illustration of the process window, the melting temperature $T_{m}=186.5\;{}^{\circ}C\;$from differential scanning calorimetry (DSC) experiments [20] with 10 K/min for PA12 (PA 2200 Balance 1.0, EOS GmbH, Krailling, Germany) and a typical temperature for the powder reservoir of $T_{p}=175\;{}^{\circ}C$ are indicated in the figure. The equilibrium melting temperature for PA12 was estimated by Dupin [6] to be$\;T_{m}^{0}=190.7\;{}^{\circ}C$ and the recrystallization temperature for a cooling rate of 10 K/min was found to be 145.5 °C [20].

The characteristic times in Figure 9 are the Frenkel time $\left(\tau_{Frenkel}\right)\;$for a pore to disappear by neglecting the gas pressure in the pore [4], the half-time ($t_{1/2}^{coal}$) for the coalescence of two individual particles from [4], the terminal relaxation time (λ) of the viscoelastic spectrum of the melt [6], the half-time of crystallization [6] and a characteristic gelation time due to chain lengthening, branching and/or cross-linking by thermal aging $t_{gel}$. $t_{1/2}^{coal}\;$for particle coalescence are estimated from the time dependence of the neck radius between particles from [4]. Chain lengthening in PA12 is due to a post-condensation reaction (see [6,20] and references therein).

Since the coalescence of polymer particles takes place for semi-crystalline materials at temperatures above their melting point and well above their glass transition temperature, the viscoelastic relaxations in the melt are highly relevant (see Section 3.3, above). For this reason, the terminal relaxation time as a representative of the longest relaxation time of the viscoelastic spectrum is also plotted in Figure 9 as a function of inverse temperature. The data are taken from frequency-dependent rheological measurements from Dupin [6,23] on PA12. They show an Arrhenius-type temperature dependence, which is typical for polymer relaxations well above the glass transition temperature.

$\tau_{Frenkel}=2\;r_{0}\;\eta /\sigma \;$is the characteristic time for a pore to disappear $\left(r_{p}=0\right)\;$in Frenkel´s late stage model, assuming a pore embedded in a Newtonian fluid and neglecting the gas pressure within the pore. For its calculation, a value for the surface tension σ for PA12 (Innov Polyamide 12, Exceltec) of 25 mN/m [6] was taken. This value was estimated from measurements from 300 to 395 K and then extrapolated linearly to process temperatures [6]. Wudy et al. referred to a value for the surface tension of PA12 (Evonik) in the melt of approximately 35 mN/m [25]. A literature survey [16] yields a value between $1\cdot 10^{-1}\;$and $5\cdot 10^{-2}N/m$ for different polymers, such as polyolefines or polystyrene, and for different gases. For η, temperature-dependent values of the zero-shear viscosity $\eta_{0}$ for PA12 were taken again from Dupin [6]. The $\eta_{0}\;$values are estimated in [6] from shear-rate-dependent data fitted by the Carreau–Yasuda equation [26]. At sufficiently high temperatures above the glass transition, the viscosity of most polymer melts follows—similar to the terminal relaxation time—an Arrhenius dependency. The activation energy was found to be $E_{a}=99\;\mathrm{kJ}/\mathrm{mol}$ [6]. In [27] for PA12 (Vestamid L-1700, Evonik, Marl, Germany) with a lower molecular weight an activation energy of $E_{a}=32.5\;\mathrm{kJ}/\mathrm{mol}$ was determined by a strain sweep test at 1 Hz in the linear viscoelastic regime. Related to the lower molar mass, the viscosity at 210 °C in this study [27] was 125 Pa·s compared to 3805 Pa·s in [6]. As stated in Section 3.3, it is difficult to define the initial pore radius $r_{0}$, and reliable experimental data could not be found. As a rough estimate, the average particle size, which is about 100 μm (see, e.g., [6,22]), was taken to calculate $\tau_{Frenkel}$. Since the spherical inclusions are formed by the gas entrapped in the intercepts between particles, the individual radius will depend on the particle size and shape distribution as well as the packing geometry. Therefore, the average pore radius is expected to be smaller than the particle radius—in the order of 10 µm—with a broad size distribution. Thus, it is obvious that the estimation of $\tau_{Frenkel}$ using $r_{0}=100\;\mu m$ and the values from [6] in Figure 9 are a rough approximation. As shown by our model, the inclusion of viscoelasticity, trapped gas and its diffusion into the matrix extends this time significantly. On the other hand, during the coalescence of all grains in the powder bed, the air present between the grains may escape through even open paths to the surface of the powder bed [21]. This process is expected to be much faster than predicted, by gas transfer into the matrix, sorption and diffusion.

Based on Frenkel´s late and early stage models [3], the times for particle coalescence and viscous pore collapse, neglecting gas transport, are in the same order of magnitude. Even without gas in the pore [3] the early stage of particle coalescence is expected to be somewhat faster than pore collapse, which is further slowed down by viscoelastic flow and gas diffusion. For illustration, a half-time for particle coalescence $t_{1/2\;}^{coal}$ using experimental data from the monitoring of the coalescence of two individual particles by Dupin [6] is plotted in Figure 9. As expected, the characteristic times for two-particle coalescence are faster than those proposed by Frenkel for viscous pore collapse. The semi-empirical viscoelastic model by Mazur and Plazek [28] combining recoverable creep compliance and the viscosity of the melt proposed even shorter times. Dupin [6] showed that the coalescence of the polymer grains is very fast (a few seconds), relative to the holding time in the polymer melt (a few tens of seconds) in the SLS process.

In Figure 9, $t_{1/2\;}^{cryst}$ is the half-time of the crystallization from isothermal crystallization experiments [6,23]. Since crystallization and coagulating kinetics during laser sintering are coupled non-isothermal processes within a nonhomogeneous temperature profile and a complex flow field, the straight line in the activation diagram represents, again, a rough estimate of the temperature and time regime. To provide a deeper insight into the effects of processing conditions on the development of microstructure during laser sintering, studies with sufficient temporal and spatial resolution are required. Whereas for sintering of the powder the particle coalescence and pore collapse are the relevant processes, for solidification of the manufactured parts before extraction, the chamber is slowly cooled down and crystallization becomes the relevant mechanism. In contrast, during the layer-by-layer building process the surface of the part bed is heated to a temperature close to the melting point in order to reduce uncontrolled crystallization and thus warping or deformation of the parts. One can introduce a modified Deborah number for the characteristic crystallization time with the pore collapse as the reference time scale: $De^{cryst}=t_{1/2\;}^{cyst}/\tau_{Frenkel}$. For this convention $De^{cryst}$ has to be well above unity, if the unfinished parts are still in the chamber, and well below one during cooling to allow solidification. The crossover from the dominance of sintering (particle coalescence and pore collapse exhibit a similar time scale [3]), on the one hand side, and the dominance of crystallization is indicated in Figure 9 by the crossing of the extrapolated lines for particle coalescence or pore collapse with the line for half-time of crystallization, on the other side.

It is well established that if polymer materials are maintained in a high-temperature environment they will degrade. Usually there is an interplay of crosslinking or chain branching and chain scission. In the case of many commercially available PA12 materials used for laser sintering, a post-condensation reaction becomes dominant at the temperatures of the powder bed, which increases molecular weight and thus increases the viscosity and influences the crystallization of the unfinished parts. The rheological properties show a gelation-like behavior which is attributed to chain lengthening (due to the post condensation), branching and/or cross-linking (see [6,20] and references therein). Assuming again the sintering (expressed by the Frenkel time) to be the relevant time scale of the process, another Deborah number for the time scale of gelation may be introduced: $De^{gel}=t_{gel}/\tau_{Frenkel}$. $De^{gel}$ has to be sufficiently large to avoid changes in the chemical structure and the resulting rheological properties, and of the mechanical performance of the final parts.

These considerations, which are exemplarily carried out for PA12, show the necessity of the measurement of experimental data under realistic conditions at or close to the processing conditions of relevant polymers in order to obtain reliable data for the comparison of experiment and theory, as well as input quantities for modeling. An extended literature survey in [16], including, in addition to PA12, other polymers such as polyethylene, polypropylene or polystyrene, yields rough estimates for the characteristic values of our viscoelastic model. Based on this literature survey, the reduced parameters can be estimated as $\tilde{\lambda }\approx 10^{-3}\;$and ${\tilde{r}}_{p,eq}\approx \;10$. If these estimations can be confirmed by more precise measurements, the gas diffusion becomes an even more relevant mechanism for the modelling of pore collapse. A value of ${\tilde{r}}_{p,eq}$ larger than unity would not permit a volume reduction in the pore by surface tension and viscoelastic flow alone, and gas diffusion would be needed [16]. Due to large uncertainties in the estimates for each single parameter, an uncertainty of more than a factor of ten is feasible and has to be taken into account for interpretation. However, if these values are close to the real ones, bubbles in polymer sintering might initially grow after densification and would only slowly disappear by diffusion-controlled phenomena. This would correspond to the surface-energy-driven viscoelastic collapse in regime III, which is allowed to happen because of the lowered bubble pressure. Liu [21] (including results from [6]) reported that, for an unsaturated polymer, the time for the disappearance of inclusions (“bubbles”) with a radius of 10 to 265 μm varies from a few milliseconds to fifty seconds. Liu further reported that surface tension had little influence in that case. For a saturated polymer, Liu observed much longer times of 10 to 90 min for the same bubbles. For the latter case, surface tension is assumed to govern the kinetics of the disappearance time of bubbles. The large time differences for the disappearance of gas bubbles [21] cannot be attributed to surface tension and viscoelasticity alone. Thus, the evolution of the size of closed pores in the material can only be explained by the diffusion and absorption of the gases in the surrounding molten polymer. Furthermore, the size and shape distribution of the particles and the resulting gussets or inclusions, temperature differences in powder bed and flow-field inhomogeneities, as well as the layer-by-layer processing and gas escape through open paths has to be included. There are a number of advanced finite element simulations (FEM) (see, e.g., [21,29]) which allow the inclusion of more of these details than in an analytical model. There are even attempts to combine FEM and machine learning techniques to evaluate the complexity of powder bed fusion and optimize additive manufacturing process parameters [30]. An analytical model is however still useful to gain insight into the general dependencies, the control variables and the understanding of the underlying processes.

## 5. Conclusions and Outlook

Based on the sintering theory introduced by Frenkel [3], a model for the pore collapse, which is the late stage of sintering of an array of polymer particles, has been developed. For this late stage, the dynamics of a gas pore in a viscoelastic melt is modeled. The viscoelasticity of the melt is represented by the convected Maxwell model. The crucial parameters of the viscoelastic pore collapse model are: the viscoelastic relaxation time λ, the ratio of surface tension and the product of initial radius and viscosity$\;\sigma /\left(r_{0\;}\eta \right)$ and the ratio of surface tension and the product of initial radius and pressure$\;p_{0}r_{0}/\sigma$. The first two parameters determine the time scale of the pore collapse dynamics, while the latter determines the equilibrium radius at long times for an idealized pore without gas transfer to the surrounding melt.

As an extension of the viscoelastic model, gas transport into the polymer matrix was included by gas diffusion. The latter is approximated by a gas transfer rate. This model extension leads to a complete collapse of the pore at long times. The time-evolution of the pore collapse and its dependence on surface tension, viscosity and gas transfer coefficient becomes rather complex. Three time regimes of pore collapse could be distinguished: surface-tension-driven regime (I), diffusion-controlled regime (II) and surface-tension-driven collapse (III). A parameter study showed that the occurrence and length of these time intervals depend on the actual parameter sets and that not all parameter sets yield all regimes.

Finally, some limitations of the proposed model with respect to polymer sintering are discussed. The different time and temperature regimes of the process steps of selective laser sintering are exemplarily depicted, described and discussed for polyamide 12 as a typical polymer for laser sintering. It becomes obvious that, although embedded gas pores are problematic for the final mechanical and optical performance of sintered polymeric parts or coatings, the pore collapse is one of many mechanisms that are linked together in a highly complex manner by the process steps of SLS or other polymer sintering processes.

Future works should first concentrate on a similar study of the parameter-dependent behavior for the solution of the full diffusion problem to validate the predictions of this approximate model. The next steps should include the solution without a quasi-steady flow approximation and the use of more elaborate rheological models, potentially including aging effects, which have been shown to be of importance in technical applications [6,23]. It would also be interesting either to model the intermediate stage not modeled in the original Frenkel model, or to consider the effect that multiple gaseous pores in a melt have on each other. Another necessity is the measurement of experimental data under realistic conditions, which we have not been able to find published. This includes the measurement of parameter values at processing conditions for relevant polymers.

## Data Availability

The data presented in this study are available on request from the corresponding author.

## Appendix A

Neglecting the convective term $\frac{d{\tilde{r}}_{p}}{d\tilde{t}}\frac{{\tilde{r}}_{p}{}^{2}}{{\tilde{r}}^{2}}\;\frac{\partial \tilde{c}}{\partial \tilde{r}}$ in the diffusion equation (Equation (17)), which is consistent with the quasi-steady flow approximation in Section 2.2 and motivated by [9], the problem can be solved analytically following a method from one-dimensional heat conduction calculations for semi-infinite one-dimensional media which can be found, e.g., in the books by Carlslaw and Jaeger [31,32] (§2.4, §2.5). Details can be found in [16]. The full solution formula for the concentration profile reads:

(A1) $$\begin{array}{l} \tilde{r}\tilde{c}=\frac{2}{\sqrt{\pi }}\overset{\infty }{\underset{\left(\tilde{r}-1\right)/2\sqrt{d\tilde{t}}}{\int }}{\tilde{K}}_{h}\tilde{p}\left(\tilde{t}-\frac{{\left(\tilde{r}-1\right)}^{2}}{4d\mu^{2}}\right)\exp\left(-\mu^{2}\right)d\mu \\ +\frac{1}{2\sqrt{\pi d\tilde{t}}}\overset{\infty }{\underset{0}{\int }}\frac{1}{s+1}\left\{\exp\left(-\frac{{\left(\left(\tilde{r}-1\right)-s\right)}^{2}}{4d\tilde{t}}\right)-\exp\left(-\frac{{\left(\left(\tilde{r}-1\right)+s\right)}^{2}}{4d\tilde{t}}\right)\right\}ds \end{array}$$

In this solution, $d=2D\eta /(r_{0}\sigma )$ is the dimensionless diffusion constant of Fickian diffusion (with D being the diffusion constant itself).
